# On the formation of Dodd-Frank Act derivatives regulations

**DOI:** 10.1371/journal.pone.0213730

**Published:** 2019-03-25

**Authors:** Shawn Mankad, George Michailidis, Andrei Kirilenko

**Affiliations:** 1 Department of Operations, Technology, and Information Management, Cornell University, Ithaca, NY, United States of America; 2 Department of Statistics, University of Florida, Gainesville, Florida, United States of America; 3 Department of Finance, Imperial College, London, United Kingdom; Centre for Economic and Regional Studies of the Hungarian Academy of Sciences, HUNGARY

## Abstract

Following the 2007-2009 financial crisis, governments around the world passed laws that marked the beginning of new period of enhanced regulation of the financial industry. These laws called for a myriad of new regulations, which in the U.S. are created through the so-called notice-and-comment process. Through examining the text documents generated through this process, we study the formation of regulations to gain insight into how new regulatory regimes are implemented following major laws like the landmark Dodd-Frank Wall Street Reform and Consumer Protection Act. Due to the variety of constituent preferences and political pressures, we find evidence that the government implements rules strategically to extend the regulatory boundary by first pursuing procedural rules that establish how economic activities will be regulated, followed by specifying who is subject to the procedural requirements. Our findings together with the unique nature of the Dodd-Frank Act translate to a number of stylized facts that should guide development of formal models of the rule-making process.

## Introduction

Financial crises induce large societal costs in the form of direct bailouts or through slower economic growth as a consequence of firm and household debt reduction [1]. Both costs were borne by the public in the aftermath of the 2007 financial crisis [2]. Accordingly, driven by public outcry, governments around the world responded with stricter regulatory frameworks. The European Union introduced a number of supervisory bodies (European Banking Authority, European Securities and Markets Authority, etc.) and internationally the Financial Stability Board was created with the mandate of promoting international financial stability. In the United States, enhanced oversight and regulation was introduced through the landmark Dodd-Frank Wall Street Reform and Consumer Protection Act. This new regulatory framework marked the beginning of a new epoch of greater regulation [3] and was proclaimed by lawmakers and then President Barack Obama as “a sweeping overhaul of the financial regulatory system, a transformation on a scale not seen since the reforms that followed the Great Depression” [4, 5]. Indeed, the Dodd-Frank Act covered a vast array of topics in its 540 sections [6].

In this paper, we focus on the pattern of new regulations created by the U.S. Commodity Futures Trading Commission (CFTC) as a result of the Dodd-Frank Act. The CFTC is a federal regulatory agency that became the main U.S. federal regulator of derivatives markets in 2010 through Title VII of the Act. Note that over-the-counter derivatives known as swaps were largely associated with the crisis and essentially unregulated in the United States, Asia and Europe [5, 7]. As such, the CFTC was faced with implementing key sections at the very core of this new regulatory regime, and did so in subsequent years through a myriad of new rules and regulations that apply to the financial industry.

The process to create federal regulations in the U.S. must adhere to a federal statute called the Administrative Procedures Act (APA), requiring that rules are developed through a three-step process. An agency must: (1) provide notice to the public of the proposed rule by publishing it in the Federal Register; (2) give the public an opportunity to provide feedback on the proposed rule; and (3) publish the final rule in the Federal Register. The public typically provides feedback in the form of “comment letters” which are submitted to a federal regulatory agency by mail or online and displayed in a dedicated online public folder on the agency’s website. Any member of the public can submit a comment letter, and often public comment letters come from a mix of interested citizens, academics, and industry participants who submit letters in their own name, through an industry association, or through their outside legal counsel. After the comment period closes, the agency weighs the overall evidence, supplied by its own data and reasoning, as well as that from the public, to ensure that its proposed regulation will accomplish the stated goal in the most effective manner. If the public commentary contains persuasive new data or policy arguments, the agency may decide to abandon the rule or modify aspects of the proposal to reflect these new issues. If an agency creates a rule without properly taking into account public commentary, the rule can be challenged and nullified through judicial review. As such, the APA allows for the pubic, broadly defined, to systematically influence the development and implementation of regulations.

The general topic of regulation has been addressed by various communities, where some key issues investigated include the role of public comments on influencing final rules [8–11], whether excessive procedural and bureaucratic constraints due to the APA create significant delay of regulation formation (the so-called “ossification theory” [12, 13]), and whether regulatory agencies speed up or slow down creation of regulations strategically according to the political environment [14]. With few recent exceptions [15–17], previous empirical analyses of the public comment rule-making process utilized surveys, interviews of comment writers and/or government workers, or teams of individuals to manually code several proposed and final rules and their corresponding comments in order to quantitatively investigate whether and how rules evolved. Due to limitations of these labor intensive processes, studies have analyzed less commented on rules or a subset of comments. For instance, recent work searched the preamble of final rules for responses to comments instead of the actual comment letters [18], made conclusions regarding high comment volumes on only 3 rules [19], excluded rules with over 2,500 comments because “data collection was too burdensome” [20], studied rules receiving less than 200 comments [8, 9, 21], and randomly sampled 10% of comments per rule [22]. These methodologies share the common drawback of potential sampling bias. For instance, simple random sampling of comments can result in sizable bias for proposals that receive a highly skewed set of comments. Such bias can also occur with respect to regulations, since highly salient rules are often avoided in empirical studies.

Note that given the quickly evolving political landscape and the nature of the Dodd-Frank Act, which represented a generational and “sweeping overhaul of the financial regulatory system” [4, 5], the CFTC moved with speed to implement new rules by putting forth 157 proposals that received over 30,000 public comments. While examining the dynamics of regulation creation, to avoid sampling bias and resource constraint issues that arise when considering only a subset of documents or manual annotation, we utilize a combination of statistical, econometric, and unstructured data analysis techniques to perform an event history analysis. Specifically, we investigate the order and rate at which new regulations at the CFTC were proposed and finalized, and the impact of public commentary on this process, to gain insight into the potential strategic behaviors of the government and its regulated constituents in creating new financial regulations following a major event like the financial crisis and landmark Dodd-Frank Act. In this sense, this study addresses a novel topic and provides empirical facts that can help guide theoretical models of regulation formation. For example, to our knowledge, we are the first to find evidence that the CFTC acted with foresight by quickly implementing critical procedural rules that establish *how* activities will be regulated, that when combined with future definitional rules extend the regulatory boundary by specifying *who* is subject to the procedural requirements.

## Results

### Univariate analysis

Starting from July 2010 when the Dodd-Frank Act was passed to August 2013, the CFTC issued 157 proposed rules, 125 of which (79%) were finalized as of April 2016 through 73 final rules. Note that multiple proposed rules can be combined into a single final rule. For example, 77 FR 41940, 77 FR 47169, and 77 FR 50425 are proposed rules about exempted entities from new clearing swap clearing rules that were combined into a single final rule 78 FR 21749. Table 1 summarizes each rule-making attempt, with several text-based attributes based on dictionaries for general sentiment, financial sentiment, litigiousness, and uncertainty (the notion of imprecision and financial risk). Table 2 shows the most frequent occurring words along these dimensions within each set of documents. We can see strong consistency in word usage between the proposed and final rules. The commentary includes different language; for instance, the words “manipulation”, “volatility”, “stability” appear often in the commentary but not in the rules. For further methodological details on the text-based measures, see the Methods section below.

**Table 1:** Summary statistics for proposed and finalized rules, and the public commentary. The reported statistics for public comments are averages over the number of proposed rules.

Statistic	N	Mean	St. Dev.	Min	Max
Proposed Rule Word Length	157	11,253.550	17,671.040	71	120,787
Proposed Rule Focus	157	0.563	0.267	0.166	0.985
Proposed Rule Sentiment Social Media	157	0.017	0.016	−0.042	0.058
Proposed Rule Sentiment Finance	157	−0.012	0.015	−0.122	0.010
Proposed Rule # Litigiousness Words	157	665.325	1,076.403	2	7,144
Proposed Rule # Uncertainty Words	157	246.153	467.076	0	4,209
Number Comments	157	197.917	1,150.263	1	14,173
Avg Comments’ Word Length	157	1,062.268	658.014	9.000	4,347.887
Avg Comments’ Focus	157	0.001	0.004	0.0001	0.042
Avg Comments’ Sentiment Social Media	157	0.020	0.013	−0.021	0.054
Avg Comments’ Sentiment Finance	157	−0.005	0.008	−0.049	0.020
Avg Comments’ # Litigiousness Words	157	51.554	32.117	0.000	189.909
Avg Comments’ # Uncertainty Words	157	24.636	16.336	0.000	102.319
Final Rule Word Length	73	25,329.900	33,109.620	150	188,224
Final Rule Focus	73	0.525	0.233	0.155	0.959
Final Rule Sentiment Social Media	73	0.016	0.019	−0.030	0.085
Final Rule Sentiment Finance	73	−0.005	0.006	−0.022	0.014
Final Rule # Litigiousness Words	73	1,373.918	1,691.310	6	7,426
Final Rule # Uncertainty Words	73	628.096	925.126	0	5,101

**Table 2:** Ten most frequent words for different dictionary-based text measures.

Text Measure	Proposed Rules	Final Rules	Comments
General Sentiment	appropriate, respect, available, cleared, benefits, effective, risk, limits, burden, oversight	appropriate, respect, cleared, benefits, available, risk, concerns, limits, burden, hedge	important, support, reform, effective, meaningful, limit, risk, excessive, speculative, manipulation
Financial Sentiment	effective, greater, transparency, integrity, benefit, burden, default, conflicts, question, disciplinary	effective, better, benefit, able, greater, default, burden, argued, concerns, conflicts	effective, transparency, stability, opportunity, best, excessive, manipulation, concerned, conflicts, volatile
Litigiousness	contract, will, rule, shall, regulation, contracts, regulatory, further, request, release	rule, will, shall, regulation, contract, regulatory, further, contracts, release, statutory	will, rule, law, contracts, regulatory, bona fide contract, regulation, further, legislative
Uncertainty	may, risk, could, exposure, believe, possible, approximately, anticipates, might, speculative	may, risk, believe, could, risks, suggested, exposure, possible, variation, revised	risk, speculation, speculative, may, believe, risks, could, possible, volatile, depend, volatility

**Fig 1:**
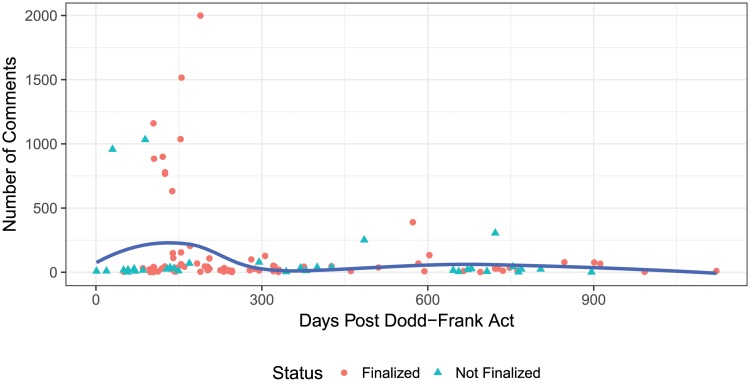
Number of comments received by the CFTC for proposed rules over time. One rule that received over 9,000 comments is plotted at 2,000 for visual interpretability. The solid line is the local average computed using a loess smoother.

From Table 1 we see that on average a rule more than doubles in length when moving from proposed to finalized, and becomes more litigious. We also see that rules generally contain negative financial sentiment, and are written in legalese, with nearly 5-10% of words signaling litigiousness or uncertainty. Each proposed rule receives on average 200 comments, though this varies widely with a rule receiving only a single comment, and as shown in Fig 1, six rules receiving over 1,000 public comments. The average focus values for all rules and comments are reported in Table 1, where we see that the average comments focus is near zero indicating lack of consensus and varied discussion.

**Fig 2:**
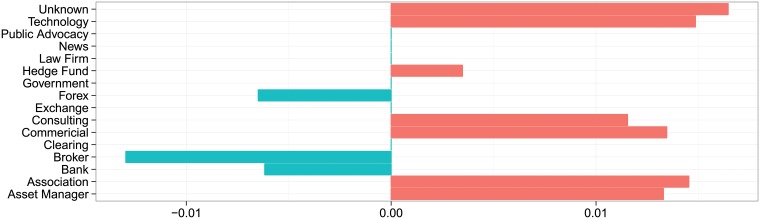
Sentiment of comments submitted by interest groups for proposed rule 75 FR 3281, which introduced a number of new requirements for registration, disclosure, recordkeeping, financial reporting, minimum capital, and other operational standards with respect to retail foreign exchange (forex) transactions. The rule was proposed on January 20, 2010, received over 9,000 comments, and was finalized on September 10, 2010 in the Federal Registar under 75 FR 55410.

In response to the 157 proposals, the agency received approximately 30,000 public comment letters that discuss the regulatory measures. In addition to being numerous, the public comments also appear very heterogeneous as shown in Fig 2 for an exemplar rule. Fig 1 shows a number of proposed rules received several hundred to thousands of comments shortly after the Dodd-Frank Act was signed into law. Using these larger comment numbers as a proxy for the economic importance or controversial nature of a rule, there is descriptive evidence that the CFTC put forth its most politically sensitive rules within the first year of the Dodd-Frank Act being signed into law.

We can also use the structure of the Dodd-Frank Act to guide our understanding of the rule’s content, as Title VII of the Dodd-Frank Act mandates that definitions be created regarding swaps. Moreover, rules that create definitions are indicated as such in their title and rule summary (the rule’s official preamble); we consider a rule as procedural if it is not definitional or establishes financial standards, data and reporting requirements, and so on. Our categorization is identical to the CFTC’s, which can also be found at https://www.cftc.gov/LawRegulation/DoddFrankAct/Rulemakings/index.htm. Data in the Supporting Information (S1 File) also includes a column indicating whether each proposed rule is procedural or definitional.

**Fig 3:**
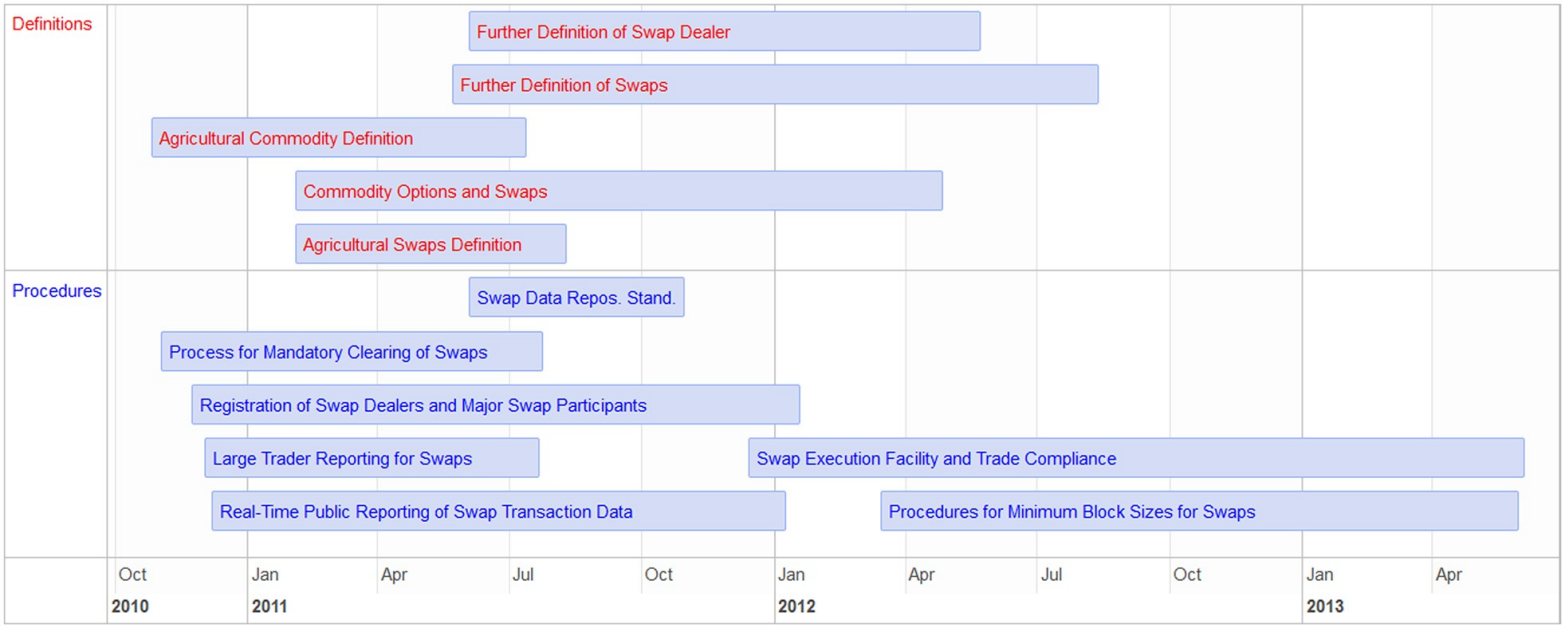
Timeline of rule-making at the CFTC for swaps. Each event marks the introduction of a proposed rule in the Federal Register to the publication date of its final version.

Fig 3 shows all definitional rules proposed by the CFTC, where it is evident that definitions were clarified after several major procedural rules that relied on this definition were proposed or even already finalized. In fact, we find that the majority of rules proposed in the first year are procedural rules that depend on foundational constructs that are undefined at the time but specified in future rule-makings. Consistent with the level of public feedback, rules in the first year tend to change more from their proposed to final form. Thus, it appears the government acted with foresight by quickly implementing critical procedural rules that are combined with future definitional rules to extend the regulatory boundary.

Table 3 shows public comments aggregated by type of author, which we manually categorized using self reported organization and name. Specifically, “Sellside” consists of providers of financial services (bank, associations and law firms writing on behalf of financial service providers), “Buyside” consists of regulated financial users of financial services (asset managers, hedge funds, associations representing and law firms writing on behalf of financial service users), “Market” consists of regulated facilitators of market and intermediation services (brokers, clearing houses, Forex, exchanges, associations representing and law firms writing on behalf of such parties), “Commercial” consists of non-financial end-users, “Retail” consists of individuals and other (actual and potential) users, “Expert” consists of non-users (government, law firms (but no client mentioned), public advocacy, consulting, technology, academic, and so on), and finally “Other” is the remainder. We find that the Market group commented on the most rules, whereas other groups like Retail, participated formally in the rule-making process only about half of the time.

**Table 3:** Frequency of comments from different segments.

Segment	Number Comments	Number Rules Commented On
Buyside	1081	121
Commercial	1120	114
Expert	1455	130
Market	1830	143
Other	22107	157
Retail	2787	78
Sellside	693	103

**Fig 4:**
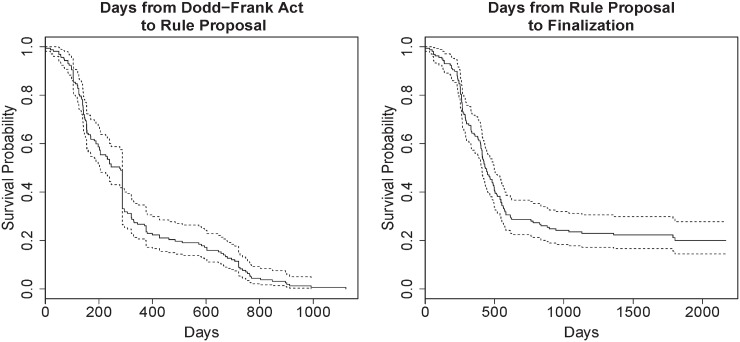
**Kaplan-Meier survival curves for two events:** (i) rule proposal and (ii) rule finalization. For (i) event times in days are defined using the Dodd-Frank Act passage as day zero. For (ii) an event time is measured in days starting with the rule proposal date. Note that some rules are never finalized.

We are ultimately interested in studying how the features above relate to the rate of two events, rule proposal and rule finalization, to gain insight into the potential strategic behavior of the CFTC in rule-making. Specifically, we aim to understand the “survival times” that are computed by counting the days from when the Dodd-Frank Act became law to when rules were proposed, and the number of days until a proposed rule becomes finalized. Fig 4 shows corresponding Kaplan-Meier survival curves. The figure shows a structural break and two regimes for both variables: a flurry of initial activity, followed by a more gradual and less volatile period later. This pattern is markedly different than what one would expect given a uniform random process which would have exhibited a smooth decreasing pattern.

**Fig 5:**
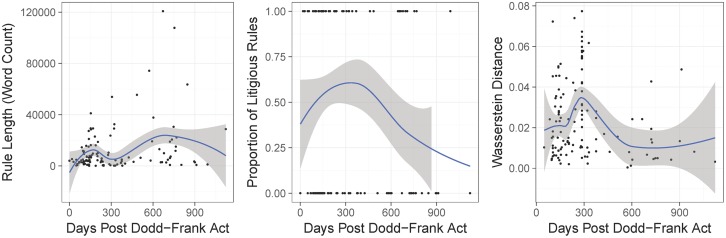
Properties of proposed rules over time. The left panel shows word count, central panel shows the percentage of highly litigious rules (defined as having a greater percentage of litigious words compared to the median level), and right panel shows the Wasserstein distance between the proposed and final rule. The solid line is the local average computed using a loess smoother.

The left and center panels of Fig 5 shows rules in the first sub-period tend to be shorter and more litigious. In fact, we find that rules in the first 400 days are approximately 8000 words long on average, including 540 litigious and 170 uncertain words. Rules proposed after the first 400 days are approximately 22,000 words long on average, including almost 1000 litigious and 500 uncertain words. To test this pattern more rigorously, we show in Table 4 statistical evidence via a regression framework that indeed the the first sub-period contain rules that are shorter, more ambiguous, and are associated with a greater amount of public commenting.

**Table 4:** Regression results testing whether proposed rules were shorter and more litigious during the first 400 days following passage of the Dodd-Frank Act. A litigious rule is one when it has a greater percentage of litigious words compared to the median level and zero otherwise.

	*Dependent variable*:		
	Rule Length	Litigious Rules	
	*OLS*	*OLS*	*logistic*
	(1)	(2)	(3)
Dummy(≤ 400 Days)	−12,702.420*** (3,204.450)	0.265*** (0.093)	1.104*** (0.406)
Constant	21,034.310*** (2,813.173)	0.306*** (0.082)	−0.821** (0.362)
Observations	157	157	157
R^2^	0.092	0.050	
Adjusted R^2^	0.086	0.043	
Log Likelihood			−104.831
Akaike Inf. Crit.			213.661
Residual Std. Error (df = 155)	16,879.040	0.491	
F Statistic (df = 1; 155)	15.713***	8.080***	

Note:

*p<0.1;

**p<0.05;

***p<0.01

We are also interested in studying how rules evolved over time when becoming finalized, especially with respect to the sub-periods discovered above and the public commentary. As such, we compute the Wasserstein distance [23] of final and proposed rule texts using the topic-document loadings from the jointly estimated LDA topic model on the text of all rules. The right panel of Fig 5 shows the rule changes more dramatically in the first sub-period after the Dodd-Frank Act became law.

In summary, data exploration has uncovered a number of interesting patterns. First is a structural break in the rule-making process, where the number of comments and several rule features exhibit interesting trends in the first year post-Dodd-Frank. For example, rules were more litigiousness in this first period, and generally became more so as they were finalized. Looking closely at the Swaps timeline in Fig 3, the level of litigiousness could be due to the first phase of expanding the regulatory boundary in which the CFTC proposed procedural rules that referenced undefined constructs to be defined later. This first phase may also drive up public commentary, since the level of uncertainty for those potentially impacted by the new regulations would be heightened until the definitional rules are completed. As such, we expect the length and litigiousness of a rule to be important features in explaining rule proposal and subsequent number of comments. We also see evidence that different segments of the public have different utility functions and strategies for influencing proposed regulations. According to extant literature, we expect interest groups to be more successful at influencing regulation when they are having high average focus, indicating highly specific and unified comments. Finally, we see that rules change more in their content from proposal to finalized during the first sub-period. We are interested in testing whether this trend holds even controlling for the proposed rule features and public comments, since this result would provide evidence of learning or strategic behavior by the CFTC.

### Multivariate analysis

To more formally test the patterns found above, we estimate a Cox proportional hazards model, where the event time is measured in days post-Dodd-Frank to rule proposal. Next through a negative binomial regression to account for overdispersion, we link these rule characteristics to the frequency of public commentary, that is, we find that certain types of rules are more likely to elicit public feedback controlling for all other features. We estimate a second Cox proportional hazards model, where the event time is days from rule proposal to rule finalization in order to understand how public comments impact rule finalization. Finally we estimate a linear model to decompose the Wasserstein distance between the final and proposed rules as a function of public commentary and timing of the rule-making action.

Table 5 shows estimation results from explaining the days to rule proposal using features derived from the text of the proposed rule itself. Two variables, the length (word count) of the proposed rule and number of litigious words, are statistically significant features. The estimated sign of the coefficients for these variables show that the CFTC was more likely to propose rules that were shorter and containing more litigious language. Combined with the trend in these variables discussed in the last section, we also know that most of the shorter and more litigious rules were proposed within the first 400 days post-Dodd-Frank.

**Table 5:** Cox proportional hazards model where the event time is number of days from the Dodd-Frank Act becoming law to rule proposal.

	Dependent variable:
	# Days to Rule Proposal
log Proposed Rule # Words	−0.00001* (0.00001)
log Proposed Rule # Litigious Words	0.299* (0.177)
log Proposed Rule # Uncertainty Words	−0.214 (0.156)
Proposed Rule Focus	0.319 (0.345)
Observations	157
R^2^	0.051
Log Likelihood	−636.195
Wald Test	7.070 (df = 4)
LR Test	8.165* (df = 4)
Score (Logrank) Test	7.201 (df = 4)

Note:

*p<0.1;

**p<0.05;

***p<0.01

Table 6 presents estimation results of a negative binomial regression to explain the number of comments submitted to each rule *i*. The goal is to try to understand whether the public was commentary decayed over time controlling for the type of rule that were proposed; therefore, we include independent variables for features derived from the text of the proposed rule and a time-trend variable that is equal to the number of days to the rule proposed from the Dodd-Frank Act being signed into law. The estimation results show that rules that contain more litigious and uncertain words tend to create more feedback from the public. Importantly, the time trend is strongly significant and negative, indicating that rules that are closer to the passage of the Dodd-Frank Act tend to have more comments.

**Table 6:** Negative binomial regression model estimation results, where the dependent variable is the number of comments submitted for a given rule.

	Dependent variable:
	# Comments
log Proposed Rule # Words	−0.694 (0.560)
log Proposed Rule # Litigious Words	0.590* (0.345)
log Proposed Rule # Uncertainty Words	0.786** (0.336)
Proposed Rule Focus	0.193 (0.396)
# Days to Rule Proposal	−0.002*** (0.0005)
Constant	4.313** (2.112)
Observations	157
Log Likelihood	−855.308
*θ*	0.587***(0.056)
Akaike Inf. Crit.	1,722.617

Note:

*p<0.1;

**p<0.05;

***p<0.01

Table 7 shows the estimation results from a sequence of nested Cox proportional hazards models, where the event time is the number of days from rule proposal to rule finalization. The first model includes only proposed rule features. We see that proposed rules are finalized at a faster rate when they are more specific (having higher focus). However, we may expect these results to suffer from omitted variable bias, since the public comments likely have an effect on rule finalization. As such, the second model additionally includes average characteristics of comments for each rule. The third model builds on the second, by additionally including the comment features averaged for each segment, which is motivated by previous literature that has shown the consensus of comments between and within segments can be particularly influential. The results show that even when accounting for public comments, rules are more likely to be finalized when they are more specific. Also longer comments with stronger sentiment reduce the rate of rules becoming finalized. We also see that certain groups providing specific commentary appear to impact the chances of rule finalization, consistent with earlier findings in political science [8, 9]. Note that Models 2 and 3, which include public commentary information, significantly improve the model fit, which confirms the importance of public feedback on the rule-making process.

**Table 7:** Cox proportional hazards model where the event time is number of days from rule proposal to rule finalization. “SM” is short for Social Media and “Fin” for Finance.

	Dependent variable: # Days to Rule Finalization		
	(1)	(2)	(3)
log Proposed Rule # Words	0.303 (0.463)	0.155 (0.524)	0.151 (0.578)
# Days to Rule Proposal	−0.0002 (0.0004)	−0.00004 (0.0005)	−0.00003 (0.001)
log Proposed Rule # Litigious Words	−0.015 (0.262)	−0.004 (0.296)	0.085 (0.334)
log Proposed Rule # Uncertainty Words	−0.306 (0.309)	−0.185 (0.382)	−0.168 (0.426)
Proposed Rule Focus	1.321*** (0.340)	1.221*** (0.357)	1.571*** (0.433)
# Comments		0.00004 (0.0001)	0.001 (0.001)
log Avg Comment # Words		−0.001*** (0.0003)	−0.001** (0.0004)
Avg Comment Focus		1.344 (0.887)	0.050 (1.417)
Avg Comment Sentiment SM		0.018** (0.009)	0.023** (0.011)
Avg Comment Sentiment Fin		−0.081*** (0.024)	−0.067** (0.026)
log Avg Comment # Litigious Words		0.161 (0.298)	0.197 (0.346)
log Avg Comment # Uncertainty Words		−0.076 (0.321)	−0.228 (0.400)
Avg Buyside Comment Focus			−1.123 (3.418)
Avg Commercial Comment Focus			−1.852 (2.625)
Avg Expert Comment Focus			3.780 (2.540)
Avg Market Comment Focus			2.002 (1.807)
Avg Retail Comment Focus			8.371* (5.024)
Avg Sellside Comment Focus			9.168*** (3.322)
# Comments by Group			Included
Observations	157	157	157
R^2^	0.099	0.206	0.272
Log Likelihood	−545.835	−535.895	−529.124
Wald Test	16.580*** (df = 5)	38.900*** (df = 12)	46.960*** (df = 24)
LR Test	16.427*** (df = 5)	36.308*** (df = 12)	49.848*** (df = 24)
Score (Logrank) Test	17.042*** (df = 5)	38.913*** (df = 12)	49.431*** (df = 24)

Note:

*p<0.1;

**p<0.05;

***p<0.01

Table 8 shows the estimation results from a similar sequence of nested linear regressions models, where the response variable is the log Wasserstein distance between the final and proposed rule. The following two variables exhibit a strong and consistent association with the outcome variable. The first is a dummy variable for the first 400 days post-Dodd-Frank. The second significant variable is average length of the submitted comment letter. Thus, even after controlling for characteristics of the proposed rule and the public comments overall and by segment, we find that the difference between the final and proposed rule content is much higher in the initial period after the Dodd-Frank Act became law, and that the CFTC tended to make fewer changes to the rule when it received longer comments.

**Table 8:** OLS regression explaining the amount change between the final and proposed rule. “SM” is short for Social Media and “Fin” for Finance.

	Dependent variable: Log Wasserstein Distance of Final and Proposed Rule		
	(1)	(2)	(3)
Dummy(≤ 400 Days)	0.869*** (0.217)	0.463** (0.221)	0.534** (0.238)
# Comments	−0.0001 (0.0001)	−0.00001 (0.0001)	0.0001 (0.001)
log Avg Comment # Words		−0.001* (0.0003)	−0.001* (0.0004)
log Avg Comment Focus		−0.494 (0.916)	−0.976 (1.301)
log Avg Comment Sentiment SM		0.013** (0.006)	0.009 (0.008)
log Avg Comment Sentiment Fin		0.010 (0.022)	0.007 (0.024)
log Avg Comment # Litigious Words		0.390 (0.242)	0.196 (0.276)
log Avg Comment # Uncertainty Words		0.348 (0.213)	0.478* (0.261)
Avg Buyside Comment Focus			−2.012 (3.626)
Avg Commercial Comment Focus			0.650 (2.346)
Avg Expert Comment Focus			1.624 (2.254)
Avg Market Comment Focus			1.573 (1.674)
Avg Retail Comment Focus			−1.761 (3.655)
Avg Sellside Comment Focus			4.955* (2.643)
# Comments by Group			Included
Intercept	Included	Included	Included
Observations	124	124	124
R^2^	0.126	0.275	0.368
Adjusted R^2^	0.112	0.224	0.246
Residual Std. Error	0.921 (df = 121)	0.861 (df = 115)	0.849 (df = 103)
F Statistic	8.730*** (df = 2; 121)	5.440*** (df = 8; 115)	3.003*** (df = 20; 103)

Note:

*p<0.1;

**p<0.05;

***p<0.01

## Discussion

Here we synthesize the full breadth of results presented above with respect to the overall rule-making process and in particular the potential strategies of the government and public. To our knowledge, we are the first in the public comment rule-making literature to uncover dynamics illustrated with the case study on swaps shown in Fig 3; Specifically, we find that the government proposed rules that had different quantitative features in the first year post-Dodd-Frank compared to later rule-makings. For instance, the rules in the initial sub-period tended to be shorter and more litigious. Combining these statistical results with a careful reading of the rules, we find that these rules also tended to create new procedures and financial operating standards for instruments and markets that were fully defined at a later date. As such, potentially driven by the heightened uncertainty from the public’s perspective around the economic significance and compliance of the new regulatory regime, public participation was also heightened in this initial sub-period as shown visually and supported by the negative binomial regression model results. While the data to understand the true motivations and causal factors leading to greater public commentary is not available, we do find anecdotal evidence that the public was alerted by the uncertainty around the new regulatory apparatus. Quoting from Prosperity Bank’s chairman and CEO, David Zalman [24], “I’ve been in banking since 1978, and today, probably over half of my time is spent with regulatory requirements. The regulatory burden is a threat to traditional community banking. It is troubling that we don’t always know what the regulators are going to want.” Industry trade publications [25] and corporate blogs [26–28] wrote about reporting requirements for swaps being finalized when the regulatory definition of swap was not yet known.

The increase in public comments had an effect on the outcome of final rules. In Table 7 we find that a greater number of longer and more pointed comments in terms of financial sentiment lower the rate of rules becoming finalized, and that certain groups (e.g., Retail and Sellside) providing specific and unified commentary appear to impact the rate of rule finalization, consistent with earlier findings in political science. For example, scholars have uncovered evidence that agencies were more likely to be influenced by sophisticated comments and that individuals raise different issues than interest groups [19], and that comments from organizations can influence regulatory outcomes, especially when industry comments are numerous and show consensus [8, 9, 29]. Though we study regulations following a major regulatory implementation, where there was necessarily a complex mixture of public interest groups that were differentially impacted and thus commented differently on the new regulations, we find evidence in support of the extant literature.

After the initial sub-period, proposed rules tended to be longer because they were often specifying complex definitions. For instance, the definition of swap was modified several times (as shown in Fig 3; see also final rules: 76 FR 49291, 77 FR 48207, 77 FR 30596.) and as a consequence the legal definition is several hundred pages long. Yet even though the specification of key definitions and constructs effectively identifies who in financial markets must comply with new operating standards, the level of public commentary was lower in the later sub-period. One explanation is that the level of uncertainty from the public’s point of view is much lower in the later sub-period, with major procedural rules already finalized. Moreover, the government may also learn from its previous rule-making experiences to make proposals in the later sub-period that expend less political capital and thus require less alteration.

We also support recent findings [13] against the so-called “ossification theory”, which proposes that over decades since the APA was first enacted, federal agencies tend to issue rules only after significant delay caused by excessive procedural and bureaucratic constraints. Counter to this theory, we find a rapid initial rate, with nearly 75% of proposed rules occurring within one year of Dodd-Frank’s passage. Once a rule is proposed, nearly the same percentage are finalized within two years—exceeding or matching rates dating back to the 1970’s [12] and more recent data published from 1983 to 2006 [13].

The narrative about strategic prioritization of certain types of rules and our corresponding findings also support recent work [14] that found regulatory agencies speed up or slow down regulations strategically according to the political environment. Note that previous work to our knowledge analyzed executive branch agencies. Here we analyze the CFTC, an independent regulatory agency that “is subject to different political pressures” [14] and follows substantially different rule-making processes. For example, the CFTC has a commission that votes on rules before they are finalized, as opposed to several stages of review by the executive branch for executive branch agencies. To our knowledge our study is one of the first to analyze the strategic behavior of such independent regulatory agencies, especially with respect to the type of rules that are pursued and following a major event like the financial crisis and landmark Dodd-Frank Act.

Our analysis has revealed interesting patterns on the process of the CFTC proposing, revising and finalizing financial regulations that implement the principles of the Dodd-Frank Act. In the rule-making process there are three parties involved: Congress that creates the statutory provision, the rule-making agency that interprets and possibly amplifies the provision, and the private agents impacted by the rules that aim to modulate their behavior. There is extensive literature both in political science and legal studies that supports the notion that bureaucrats (the rule making agency) have preferences that may or may not be aligned with those of Congress, or the private agents they regulate. Such preferences need not be ideological, but also pragmatic and implementation oriented since the agency beyond rule-making is also in charge of enforcement [10, 11, 17, 30–33]. This suggests that the agency (e.g. CFTC) can follow complex strategies in deciding when and which rules to introduce first and based on the public’s feedback how to revise them before final approval. Our findings together with the unique nature of the Dodd-Frank Act translate to a number of stylized facts that should guide development of formal models that incorporate preferences of all parties involved in the rule-making process, and also provide empirically testable hypotheses.

Another important issue in this work is utilizing large unstructured data (text) and extracting features that can be subsequently employed in standard statistical models. We believe that our methodological approach will become a necessity to study the APA rule-making process due to the increasing utilization and ease of electronic comment submission. For example, the so-called “net neutrality” rule recently proposed by the U.S. Federal Communications Commission ultimately received over 9 million public comments, though many submissions are believed to be potentially fraudulent or part of sophisticated organized campaigns [34, 35]. The problem of fraudulent comments and organized campaigns represents a key area of future work, where we believe that investigating comment arrival rates stratified by their content and sentiment will lead to interesting discoveries. Another important future work is to develop support tools from the perspective of the regulator. As the number of comment submissions grows, effective support tools that are driven by statistical modeling are necessary, so that public officials are not overwhelmed by a flood of documents to review. These are important methodological problems that can ultimately help make governments and public policy more efficient and transparent, and thus create large societal benefits [36].

## Methods

All statistical analysis was performed in R version 3.4.4 [37]. Text-analysis was performed using the “tm” [38] and “topicmodels” [39] packages. The Cox proportional hazards model was estimated using the “survival” package [40].

### Text-based measures

We calculate a number of text-based features through word counts with different dictionaries, as reported in Table 1. Specifically, we compute sentiment as the normalized sum of positive words minus the sum of negative words for each document. To summarize the tone of documents that are written more casually, we utilize dictionaries [41, 42] that were created, respectively, to summarize the opinions within online customer reviews and social media sites. In total, the combined dictionaries consist of approximately 10, 000 labeled words that are labeled by their positive or negative sentiment strength. Most comments, however, consist of economic or legal arguments. As such, we also utilize the positive and negative dictionaries developed in [43] by training on 10-K filings (annual reports) of publicly traded companies to the U.S. Security and Exchange Commission. Lastly, we utilize the litigiousness and uncertainty dictionaries from [43] to further characterize the text content with additional normalized word counts. If a document has high litigiousness score, then the document has a propensity for legal contest. The dictionary includes terms like “claimant”, “deposition”, “testimony”, and “tort”, and thus reflects a more litigious environment. Likewise, an uncertainty score closer to one means the document has more emphasis on the general notion of imprecision and financial risk, with terms like “approximate”, “contingency”, “fluctuate”, “indefinite”, “uncertain”, and “variability” in the dictionary. Thus, we calculate four features (sentiment with informal writing, financial sentiment, litigiousness, and uncertainty) that are dictionary-based; note that all dictionaries are chosen because they were developed for text generated in similar domains and contexts, which addresses a major challenge in dictionary-based analysis [44].

The last text measure that we compute is the focus or specificity of the rule or comment. Underlying our measure are probabilistic topic models, which are a popular class of algorithms in text mining that aim to automatically summarize large archives of text by discovering hidden “topics” that occur within a corpus [45].

Topic models assume that all documents share the same topic set, but each document exhibits a different mixture of those topics. A statistical model called Latent Dirichlet Allocation (LDA) tries to capture this intuition. Due to space constraints, the reader is referred to [45] for details of this generative model. We provide a formal definition of the joint distribution defined by LDA using the notation in [45]. The topics are *β*_1:*K*_ = {*β*_1_, …, *β*_*K*_}, where each *β*_*k*_ is a distribution over words. The topic proportions for the *d*th document are *θ*_*d*_, where *θ*_*d*,*k*_ is the topic proportion for topic *k* in document *d*. The topic assignments for the *d*th document are *z*_*d*_, where *z*_*d*,*n*_ is the topic assignment for the *n*th word in document *d*. The observed words for document *d* are *w*_*d*_, where *w*_*d*,*n*_ is the *n*th word in document *d*, which is an element from the fixed vocabulary.

The generative process assumed by LDA topic models defines the following joint distribution of observed and hidden variables

$$\begin{array}{ccc} p(\beta_{1:K},\theta_{1:D},z_{1:D},w_{1:D}) & = & \prod_{i=1}^{K}p\left(\beta_{i}\right)\prod_{d=1}^{D}p\left(\theta_{d}\right)\times \\ & & \prod_{n=1}^{N}p\left(z_{d,n}|\theta_{d}\right)p\left(w_{d,n}|\beta_{1:K},z_{d,n}\right). \end{array}$$

The key challenge behind topic models is to use tools like Gibbs sampling or variational algorithms [46–48] to calculate the posterior distribution *p*(*β*_1:*K*_, *θ*_1:*D*_, *z*_1:*D*_|*w*_1:*D*_). Extensive work in computer science and applied statistics has led to fast algorithms capable of analyzing extremely big text archives. For complete statistical and algorithmic details on the topic model, see [45, 49] and references therein.

In our empirical work, the topic model was estimated jointly for all proposed and final rules, with 25 topics chosen through cross-validation. A second topic model was estimated on the corpus of comments, with 50 topics chosen through cross-validation.

Using the LDA model, to quantitatively measure the spread of discourse in a single document *d*, we define focus as

$$\text{focus}\left(d\right)=\frac{H-1/K}{1-1/K},$$ (1)

where $H=\sum_{k=1}^{K}\theta_{d,k}^{2}$. In other contexts such as economics, focus is known as the normalized Herfindahl index and is bounded by 0 and 1. This is an intuitive and useful variable, since it captures information akin to the 2nd moment (i.e. variance) of the textual content. High values for focus mean that the document is concentrated on a specific topic or issue, and has low diversity in the topics of discussion, whereas lower values of focus mean multiple topics are discussed within the same document. In principle, other measures of spread like entropy or variance could be used. The above definition of focus was chosen, since it is bounded and higher values denote higher concentration.

To quantify the change between proposed and final rule texts, we compute the Wasserstein distance (also called the “Earth Mover’s Distance” [23]) using the topic-document loadings $\theta_{d_{1}}$ and $\theta_{d_{1}}$ from the jointly estimated LDA topic model on the text of all rules. The Wasserstein distance is defined as the total cost incurred when transporting probability $\theta_{d_{1}}$ to probability $\theta_{d_{2}}$ in an optimal way, where the cost of transporting a unit of mass from *x* to *y* is given as ∥*x* − *y*∥_1_. This definition captures the absolute distance in the rule-topic probabilities, assuming that each probability is assigned a point mass.

### Models

The Cox proportional hazards model is defined as follows: Let *X*_*i*_ = (*X*_1_, ⋯, *X*_*p*_) be the values of the covariates for rule *i*, such as the word count, litigiousness, and focus of the rule. The hazard function for the Cox proportional hazard model takes the form

$$\lambda \left(t|X_{i}\right)=\lambda_{0}\left(t\right)\text{exp}\left(\beta_{1}X_{i1}+\cdots +\beta_{p}X_{ip}\right).$$

Next, let *Y*_*i*_ denote the observed time (either censoring time or event time) for subject *i*. Let *C*_*i*_ be the indicator that the time corresponds to an event (i.e. if *C*_*i*_ = 1 the event occurred, e.g., a rule was proposed and if *C*_*i*_ = 0 the time is a censoring time, we don’t have information of whether a rule got finalize since we reached the end of the observation period in this study). Ignoring ties which is the case in our data, conditioned upon the existence of a unique event at some particular time *t* the probability that the event occurs for rule *i* for which *C*_*i*_ = 1 and *Y*_*i*_ = *t* is

$$L_{i}\left(\beta \right)=\frac{\theta_{i}}{\sum_{j:Y_{j}\geq Y_{i}}\theta_{j}},$$

where *θ*_*j*_ = exp(*X*_*j*_ *β*) (see [50] for details). A key property of this model is that the factors of the baseline hazard factors λ_0_() that would be present in both the numerator and denominator have canceled out and hence they do not need to be specified explicitly, which is the case for other survival models in the literature. Then, one obtains the partial likelihood to estimate the parameters of interest.

We also utilize a negative binomial regression model to explain the number of comments submitted for each proposed rule

$$\begin{array}{ccc} p(Y_{i}=y) & = & \frac{\Gamma (y+1/\alpha )}{\Gamma (y+1)\Gamma (1/\alpha )}{\left(\frac{1}{1+\alpha \mu }\right)}^{\alpha^{-1}}{\left(\frac{\alpha \mu }{1+\alpha \mu }\right)}^{y}\\ \text{log}(\mu ) & = & \text{exp}(\beta_{1}X_{i1}+\cdots +\beta_{p}X_{ip}), \end{array}$$

where *Y*_*i*_ is the number of comments submitted to rule *i*, *μ* > 0 is the mean, and *α* > 0 is the heterogeneity parameter. Even though the response variable is a count of comments submitted for a given rule, the negative binomial distributional assumption is preferred over the Poisson distribution due to overdispersion, that is, the variance of this variable is much larger than its mean (see Table 1).

## Supporting information

S1 File Data and analysis code. This zip file contains raw and processed data for the comments and rules, as well as R code to reproduce the main results presented in this paper.
(ZIP)
