# Supplementary material for: On the formation of Dodd-Frank Act derivatives regulations
Source: PLoS One. 2019 Mar 25;14(3):e0213730. doi: 10.1371/journal.pone.0213730 (PMC6433441; doi:10.1371/journal.pone.0213730)
Supplement: S1 File — This zip file contains raw and processed data for the comments and rules, as well as R code to reproduce the main results presented in this paper. (ZIP) [file pone.0213730.s001.zip › Data_Code/ProcessedData/Data_Dictionary.docx]

**Column definitions:**

1. Outcome variable: days from intro of regulation until final event passage or rejection
2. Finalized: passage (1), non passage (0)
3. Time_doddfrank: time in days of introduction of regulation since passage of Dodd frank
4. proposedRuleTextLength_numwords: Complexity measure: length of proposed regulation in # of words
5. proposedRuleText_HIndex: Complexity measure: Heirfindahl index of the P(Topic | Rule) over topics (estimated jointly with proposed and final rules)
6. proposedRuleText_sentiment_sm: Complexity measure: sentiment score with social media dictionary
7. proposedRuleText_sentiment_fin: Complexity measure: sentiment score with finance (10-K) dictionary
8. proposedRuleText_litigiousness: Complexity measure: litigiousness score with finance (10-K) dictionary
9. proposedRuleText_uncertainty: Complexity measure: uncertainty score with finance (10-K) dictionary
10. number_comments: # of comments
11. commentTextLength_numWords_avg: average length of comment
12. commentsText_HIndex_avg: Heirfindahl index of the P(Topic | Comment) over topics
13. commentsText_sentiment_sm_avg: sentiment score with social media dictionary
14. commentsText_sentiment_fin_avg: sentiment score with finance (10-K) dictionary
15. commentsText_litigiousness_avg: litigiousness score with finance (10-K) dictionary
16. commentsText_uncertainty_avg: uncertainty scores with finance (10-K) dictionary
17. buyside_comment_count: number of comments from buyside organizations
18. commercial_comment_count: number of comments from commercial organizations
19. expert_comment_count: number of comments from experts
20. market_comment_count: number of comments from market organizations
21. other_comment_count: number of comments from others (individuals, none, etc.)
22. retail_comment_count: number of comments from retails organizations
23. sellside_comment_count: number of comments from sellside organizations
24. commentsText_HIndex_avg_buyside: Heirfindahl index of the P(Topic | Rule) over topics for comments from buyside organizations
25. commentsText_HIndex_avg_commercial: Heirfindahl index of the P(Topic | Rule) over topics for comments from commercial organizations
26. commentsText_HIndex_avg_expert: Heirfindahl index of the P(Topic | Rule) over topics for comments from expert organizations
27. commentsText_HIndex_avg_market: Heirfindahl index of the P(Topic | Rule) over topics for comments from market organizations
28. commentsText_HIndex_avg_other: Heirfindahl index of the P(Topic | Rule) over topics for comments from others (individuals, none, etc.)
29. commentsText_HIndex_avg_retail: Heirfindahl index of the P(Topic | Rule) over topics for comments from retail organizations
30. commentsText_HIndex_avg_sellside: Heirfindahl index of the P(Topic | Rule) over topics for comments from sellside organizations
31. finalRuleTextLength_numwords: Complexity measure: length of final regulation in # of words
32. finalRuleText_HIndex: Complexity measure: Heirfindahl index of the P(Topic | Rule) over topics (estimated jointly with proposed and final rules)
33. finalRuleText_sentiment_sm: Complexity measure: sentiment score with social media dictionary
34. finalRuleText_sentiment_fin: Complexity measure: sentiment score with finance (10-K) dictionary
35. finalRuleText_litigiousness: Complexity measure: litigiousness score with finance (10-K) dictionary
36. finalRuleText_uncertainty: Complexity measure: uncertainty score with finance (10-K) dictionary
37. ell_infty: comparison between final and proposed rule using the topic distributions (estimated jointly with proposed and final rules) and the ell_infty norm
38. ell_1: comparison between final and proposed rule using the topic distributions (estimated jointly with proposed and final rules) and the ell_1 norm
39. ell_2: comparison between final and proposed rule using the topic distributions (estimated jointly with proposed and final rules) and the ell_2 norm
40. ell_1_threshold: comparison between final and proposed rule using the topic distributions (estimated jointly with proposed and final rules) and the ell_1 norm after thresholding values less than 5%
41. wasserstein: comparison between final and proposed rule using the topic distributions (estimated jointly with proposed and final rules) and the wasserstein (earth movers) distance
