# Supplementary material for: On the formation of Dodd-Frank Act derivatives regulations
Source: PLoS One. 2019 Mar 25;14(3):e0213730. doi: 10.1371/journal.pone.0213730 (PMC6433441; doi:10.1371/journal.pone.0213730)
Supplement: S1 File — This zip file contains raw and processed data for the comments and rules, as well as R code to reproduce the main results presented in this paper. (ZIP) [file pone.0213730.s001.zip › Data_Code/RawData/Data_Dictionary.docx]

**CommentData.xlsx**

**Column definitions:**

1. Comment URL – URL to the CFTC website
2. First Name – First name of the comment letter author
3. Last Name – Last name of the comment letter author
4. Organization – Organization of the comment letter author
5. Date – Date of the comment letter
6. Rule – Rule to which the comment was sent
7. Buyside – Binary variable indicating group membership
8. Sellside – Binary variable indicating group membership
9. Commercial – Binary variable indicating group membership
10. Expert – Binary variable indicating group membership
11. Market – Binary variable indicating group membership
12. Retail – Binary variable indicating group membership
13. Comment Text – Text of the submitted comment

**RuleData.xlsx**

**Column definitions:**

1. ProposedRule – name of rule
2. ProposedRuleDate – date proposed rule appeared in the federal register
3. ProposedRuleURL – URL to the CFTC website
4. ProposedRuleDate_integer – date in integer form for the event study analysis
5. Definitional – binary variable indicating whether the proposed rule was definitional
6. Finalized – binary variable indicating whether the proposed rule was finalized
7. FinalRule – name of rule
8. FinalRuleDate – date final rule appeared in the federal register
9. FinalRuleURL – URL to the CFTC website
10. FinalRuleDate_integer – date in integer form for the event study analysis
11. Days Since Dodd-Frank – integer indicating days from proposal to passage of Dodd-Frank
12. Days Between Final and Proposed – integer indicating days between final and proposed rule
13. FinalRuleText – text of the final rule
14. ProposedRuleText – text of the proposed rule
